# Breakfast and Breakfast Cereal Choice and Its Impact on Nutrient and Sugar Intakes and Anthropometric Measures among a Nationally Representative Sample of Australian Children and Adolescents

**DOI:** 10.3390/nu9101045

**Published:** 2017-09-21

**Authors:** Flavia Fayet-Moore, Andrew McConnell, Kate Tuck, Peter Petocz

**Affiliations:** 1 Nutrition Research Australia, Level 13 167 Macquarie Street, Sydney 2000, Australia; andrew@nraus.com (A.M.); kate@nraus.com (K.T.); 2 Department of Statistics, Macquarie University, Sydney 2109, Australia; peter.petocz@mq.edu.au

**Keywords:** cereal, breakfast, sugars, children, adolescent, nutrient, National Nutrition Survey, BMI

## Abstract

There is limited evidence in Australia that compares the nutritional impact of a breakfast cereal breakfast to a non-cereal breakfast, and includes the type of cereal. This study investigated the impact of breakfast choice and the total sugar content of breakfast cereal on nutrient intakes and anthropometric measures among Australian children and adolescents. Data from 2 to 18-year-old in the 2011–2012 National Nutrition and Physical Activity Survey were used (*n* = 2821). Participants were classified as breakfast cereal consumers (minimally pre-sweetened (MPS) or pre-sweetened (PS)), non-cereal breakfast consumers, or breakfast skippers. Foods consumed for breakfast, foods added to the cereal bowl, and the impact of breakfast choice on daily nutrient intakes and anthropometric measures were determined. Although only 9% of children skipped breakfast, 61% of skippers were aged 14–18 years. Among breakfast consumers, 49% had breakfast cereal, and 62% of these exclusively consumed MPS cereal. Breakfast skippers had a higher saturated fat intake than breakfast cereal consumers, and lower intakes of dietary fibre and most micronutrients (*p* < 0.001). Compared with non-cereal breakfast consumers, breakfast cereal consumers had similar added and free sugars intakes, lower sodium, and higher total sugars, carbohydrate, dietary fibre, and almost all other micronutrients (*p* < 0.001). The only difference in nutrient intakes between MPS and PS cereal consumers was higher folate among PS consumers. No associations between anthropometric measures and breakfast or breakfast cereal choice were found. The highest prevalence of breakfast skipping was among 14–18-year old. Breakfast cereal consumers had higher intakes of dietary fibre and most micronutrients compared with non-cereal breakfast consumers and skippers, and almost no differences were found between MPS and PS cereal consumers.

## 1. Introduction

Breakfast is an important eating occasion for children and adolescents. Children who eat breakfast are more likely to have higher nutrient intakes, make more healthful food choices throughout the day, and have a lower risk of overweight and obesity [1,2]. Breakfast intake has also been associated with reduced weight gain and chronic disease risk in longitudinal analyses [3,4]. 

In Australia, a secondary analysis of the 2007 Australian National Children’s Nutrition and Physical Activity Survey reported that the majority of children consumed breakfast, although breakfast consumption declined markedly as children aged [5]. Only 4% of children aged 2–16 years skipped breakfast, but 59% of skippers were aged between 14 and 16 years, and girls were more likely to skip breakfast than boys. Among breakfast consumers, over two-thirds of children and adolescents consumed a breakfast cereal.

A large body of evidence indicates that the consumption of breakfast cereal may be particularly beneficial. Children who eat breakfast cereal, compared to other breakfasts or breakfast skippers, generally have healthier micronutrient profiles, including higher daily intakes of vitamins A and D, the B vitamins thiamin, riboflavin, niacin, pyridoxine, and folate, and the minerals iron, magnesium, and zinc [2,5,6,7]. Breakfast cereal is a main contributor of dietary fibre in the Australian diet [8], and studies find that breakfast cereal consumers have higher daily intakes of whole grains [2]. Breakfast cereal consumption is also an important driver of milk consumption, which facilitates higher calcium intakes [2]. 

There is limited evidence in Australia comparing a cereal breakfast to a non-cereal breakfast, including the type of cereal and the foods and beverages consumed with both types of breakfasts, and how that may influence total daily nutrient intakes. The nutrient profile of breakfast cereals in Australia varies [9], with ready-to-eat cereals generally fortified with micronutrients and muesli, whereas granola and porridge not usually fortified. The role of sugar in breakfast cereals is debated: some researchers have found that the total sugars content of a breakfast cereal is unrelated to its nutrient density [9], energy density [10], and the body weight of those who usually consume it [11]. Data from the 2011–2013 Australian Health Survey show that breakfast cereals contribute 3% of both added sugars and free sugars to the diet per capita [12]. Another consideration is that a consumer may add sugar or honey to a lower sugar cereal, influencing the total sugars content of the diet. The type of breakfast cereal consumed and the foods added to breakfast cereal might therefore be important factors contributing to total nutrient intakes that needs investigation.

There is a need to determine the impact that breakfast cereal intake, non-cereal breakfast intake, and breakfast skipping has on total nutrient intakes and anthropometric measures in Australian children and adolescents. These parameters can be measured using the latest nationally representative Australian data from 2011–2012. This study investigated (1) the foods consumed with a cereal versus a non-cereal breakfast, and the types and amounts of foods added to the cereal bowl, including sugar; (2) the impact that breakfast choice has on daily nutrient intakes and anthropometric measures; and (3) the associations between the type of breakfast cereal, including its total sugars content, on total daily nutrient intakes. 

## 2. Materials and Methods 

### 2.1. Survey Methodology

Data from the nationally representative 2011–2012 National Nutrition and Physical Activity Survey (NNPAS) were used. The NNPAS was undertaken by the Australian Bureau of Statistics (ABS) as part of the 2011–2013 Australian Health Survey [13]. Private households from urban and rural areas in all of the Australian states and territories were selected for the survey with a stratified multi-stage area sampling plan. One adult and one child were randomly selected from each household to participate in the survey. Data were collected for the NNPAS during face-to-face interviews conducted by trained ABS interviewers. Between May 2011 and June 2012, 12,153 survey participants were interviewed, on all days of the week and all months of the year to account for seasonal variation. An adult was interviewed on the child’s behalf for children aged 2–14 years, however, children aged 6–14 years were invited to also participate in the interview. Children aged 15–17 years were personally interviewed with parental consent. An Automated Multiple-Pass Method, developed by the Agricultural Research Service of the United States Department of Agriculture, was used to capture all of the foods and beverages consumed by respondents during the 24 h prior to the interview day. The interviewers used set questions based on the type of food reported to gain more detail, and a Food Model Booklet [14], which included photos and drawings, to more accurately determine portion sizes. A second day of dietary recall was provided via telephone interview by approximately two-thirds of respondents. For this study, data from the 2812 respondents aged 2–18 years were examined. Two-day data were used to calculate the usual consumers of breakfast cereal. To maximise the sample size, the first day of dietary recall was used for all other analyses, and data were weighted to represent the Australian population with weightings provided by the ABS. The interview components of the survey were conducted under the Census and Statistics Act 1905. Ethics approval was not necessary.

### 2.2. Usual Consumers

Of the 2812 respondents aged 2–18 years, 1682 provided a second day of dietary recall. The Multiple Source Method (MSM)—an alternative equivalent to the National Cancer Institute method [15]—was used to estimate the usual prevalence of breakfast cereal intake. The MSM used a logistic regression model to calculate each respondent’s probability of consuming breakfast cereal on a random day. Descriptive statistics based on these probabilities were calculated to represent the intake distribution of the entire sample.

### 2.3. Breakfast and Breakfast Cereal Intake

As part of the 24-h dietary recall, interviewers asked respondents to state the eating occasion for each food and beverage consumed from 11 options: breakfast, brunch, morning tea, lunch, afternoon tea, dinner, supper, snack, beverage/drink, extended consumption, and other. This study defined breakfast as all foods and beverages consumed at the “breakfast” eating occasion, irrespective of time of day. In addition, any food from the sub-major food groups “Breakfast cereals, ready to eat” or “Breakfast cereals, hot porridge style” was classified as breakfast if it was reported as an “extended consumption” eating occasion, and was consumed between 5.30 a.m. and 9.30 a.m.

Each survey respondent was classified as a “breakfast skipper” or “breakfast consumer”, and breakfast consumers were classified as either a “non-cereal breakfast consumer” or a “breakfast cereal consumer”. Breakfast cereal consumers were further classified according to the total sugars content of the cereal.

“Breakfast cereal” was defined as any food from the sub-major food groups “Breakfast cereals, ready to eat” or “Breakfast cereals, hot porridge style” consumed as breakfast. A non-cereal breakfast consumer was a breakfast consumer who did not report having breakfast cereal, and a breakfast skipper was a respondent who did not report having breakfast.

Breakfast cereal was further classified as “minimally pre-sweetened” (MPS) if it contained <15% total sugars (<15 g/100 g), and “pre-sweetened” (PS) if it was ≥15% (≥15 g/100 g) total sugars. The ABS defined a discretionary breakfast cereal as those with >30 g/100 g total sugars or breakfast cereals with added fruit whose total sugars were >35 g/100 g [12]. For the purposes of analysing the impact of breakfast cereal sugars to added sugars and free sugars in this study, the pre-sweetened breakfast cereal category was classified into two categories: ≥15 g and <30 g sugars/100 g (PS15) and ≥30 g sugars/100 g (PS30).

A MPS cereal consumer was defined as a respondent who was an exclusive consumer of MPS cereal, whereas a PS cereal consumer was a respondent who consumed PS cereal (including those who may also have had a MPS cereal). A PS15 cereal consumer consumed PS15 cereal (including those who also consumed MPS cereal), and a PS30 cereal consumer consumed PS30 cereal (including those who also consumed MPS and/or PS15 cereal).

All breakfast cereals from the sub-major food group “Breakfast cereals, ready to eat” were defined as “ready-to-eat” (RTE), with the exception of 19 foods that had the word “muesli” in the name and were part of the minor food groups “Breakfast cereal, mixed grain, with fruit and/or nuts” and “Breakfast cereal, mixed grain, with fruit and/or nuts, fortified” were defined as “Muesli”. Breakfast cereals from the sub-major food group “Breakfast cereals, hot porridge style” were defined as “Hot porridge style”. A RTE cereal, muesli, or hot-porridge style cereal consumer was defined as a respondent who was an exclusive consumer of that type of cereal. A respondent who consumed more than one of these types of cereal was defined as a “mixed” cereal consumer, and was excluded from the statistical analyses.

For each food or beverage reported in the dietary recall, respondents stated “whether it was eaten in combination with another food”. One of the 13 options given was “cereal with additions”: any food or beverage with this combination code that was consumed at the same time as breakfast cereal was defined as an addition to the breakfast cereal bowl. The addition was defined as “sugar” if it was from the minor food groups “Sugar”, “Honey and sugar syrups”, and “Jams and conserves, sugar sweetened”. The prevalence of breakfast cereal consumers that added each sub-major food group to the cereal bowl was calculated. The prevalence of breakfast cereal consumers that added sugar to the cereal bowl and the amount added, in grams, was calculated. The top 10 sub-major food groups and the median grams consumed per consumer of each food group was calculated.

### 2.4. Nutrient Intakes

Dietary intake data were analysed using the survey specific 2011–2013 Australian Food Composition Database (AUSNUT) developed by Food Standards Australia New Zealand (FSANZ) [16]. Daily energy and nutrient intakes were calculated for all respondents. The absolute amount and proportion of daily energy and nutrient contribution from breakfast cereal was also calculated. The macronutrients analysed were protein; total and saturated fat; total, added, and free sugars; carbohydrate; and fibre. The micronutrients analysed were niacin, iron, thiamin, riboflavin, folate, calcium, sodium, magnesium, and potassium. 

The contribution to daily added and free sugars from the breakfast cereal bowl was calculated, where the breakfast cereal bowl refers to all additions to the cereal bowl, as well as the breakfast cereal. Added sugars are defined by FSANZ as all added forms of dextrose, fructose, sucrose, lactose, sugar syrups, and fruit syrups, and free sugars as all added sugars, as well as the sugars in honey, fruit juice, and fruit juice concentrates. Free sugars do not include natural sugars in intact fruits and vegetables or milk sugars.

### 2.5. Anthropometric Measures

Physical measurements including weight, height, and waist circumference were measured for all respondents by trained interviewers during the face-to-face interview. Body mass index (BMI) *z-*score, also known as BMI standard deviation (SD) score, is a measure of relative weight adjusted for age and sex. The BMI *z-*score was calculated using the child’s age, sex, height, and weight, and the World Health Organisation growth reference standards for 2–4 and 5–19-year-old children [17]. The standard normal distribution was then calculated for all children’s BMI *z-*scores. This was used to categorise children as: normal weight (<85%), at risk for overweight (≥85% to <95%), or overweight (≥95%). Each child’s waist circumference to height ratio was calculated as an additional indicator of health risks associated with obesity. In children, a waist circumference to height ratio of <0.5 is associated with a low risk of metabolic complications from obesity, whereas a ratio of ≥0.5 is associated with a higher risk [18].

### 2.6. Statistical Analyses

The statistical package IBM SPSS version 21.0 (IBM Corp., Armonk, NY, USA) was used for all analyses, and due to the relatively large sample size (*n* = 2812), *p*-values < 0.001 were treated as significant. General linear models were adjusted for factors including age, sex, BMI *z-*score, and energy intake, and ANOVA tables and chi-square tests for independence showed relationships between variables. Bonferroni post-hoc analysis showed pairwise significance between categories of consumers. 

## 3. Results

Based on usual intakes, 47% of children were usual consumers of breakfast cereal. Based on day one data, 9% of children and adolescents skipped breakfast, and 61% of those who skipped were aged between 14 and 18 years (Table 1). Over half of breakfast consumers had a non-cereal breakfast (51%).

Age group was significantly associated with the prevalence of breakfast cereal consumption (*p* < 0.001), and decreased with age, from 58% among 2–3-year-old to 32% among 14–18-year-old (Table 1). There was no significant difference in the prevalence of breakfast cereal consumption between boys and girls. Nearly two-thirds of breakfast cereal consumers exclusively had a minimally pre-sweetened (MPS) cereal, while 32% consumed a pre-sweetened (PS) cereal, and 8% had a PS30 breakfast cereal. There was no significant association between age group or sex and prevalence of breakfast cereal consumption by the total sugars content of the breakfast cereal. 

The majority (87%, *n* = 1103) of breakfast cereal consumers were exclusive consumers of a ready-to-eat (RTE) cereal. Only 1% (*n* = 18) had muesli, while 11% (*n* = 137) had hot-porridge style cereal, and 1% (*n* = 10) of participants were mixed cereal consumers (Supplementary Table S1). Daily nutrient intakes and contributions by type of breakfast cereal can be found in the Supplementary Tables (Supplementary Tables S2 and S3). Due to low n values for consumers of muesli, hot-porridge style, and mixed cereal, there were relatively few statistically significant results when comparing these consumers. All further results that sub-classify breakfast cereal consumers by type of breakfast cereal do so by the pre-sweetening of the cereal (MPS vs. PS cereal consumers).

### 3.1. Food Groups Consumed at Breakfast

89% of breakfast cereal consumers had ready-to-eat breakfast cereal and 87% had dairy milk (162 g) at breakfast. In comparison, 56% of non-cereal breakfast consumers had bread and 25% had dairy milk (206 g) (Table 2). One in 10 breakfast cereal consumers had bread and bread rolls at breakfast, with an average serving size of 35 g, compared to 54 g among non-cereal breakfast consumers. Four of the other top 10 most popular food groups among non-cereal breakfast consumers were those commonly consumed with bread and bread rolls such as margarine and table spreads, yeast extracts, butters, honey, jam and spreads; whereas only margarine was in the top 10 among breakfast cereal consumers. Waters, and fruit and vegetable juices and drinks were reported by both cereal and non-cereal breakfast consumers.

Almost one third (32%) of breakfast cereal consumers consumed the food group sugar, honey and syrups at breakfast, compared with 1 in 10 non-cereal breakfast consumers (Table 2). One in ten non-cereal breakfast consumers consumed jams and sweet spreads, compared to <2.8% of breakfast cereal consumers. 

Breakfast cereal consumers added, on average, 3.2 g ± 0.3 g of sugar to their breakfast cereal. There was no significant difference in the amount of sugar added by sex or between age groups. However, the type of cereal influenced the amount of sugar added. MPS cereal consumers added significantly more sugar to the cereal bowl (5.2 ± 0.4 g) than PS cereal consumers (0.0 ± 0.5 g).

### 3.2. Energy and Nutrient Intakes by Type of Breakfast Consumer

Breakfast skippers had a lower daily energy intake than breakfast cereal consumers, and tended to have a more undesirable nutrient profile, including the highest intake of saturated fat and the lowest intakes of dietary fibre and most micronutrients (Table 3). Compared with non-cereal breakfast consumers, breakfast cereal consumers had a 7% lower intake of total fat, and a 4% higher intake of carbohydrate, 15% higher fibre intake and 7% higher total sugars intake. Total daily intakes of added and free sugars were not significantly different between breakfast skippers, breakfast cereal consumers, and non-cereal breakfast consumers. There were no significant differences in the total daily intakes of protein, saturated fat, or niacin. Apart from niacin and sodium, breakfast cereal consumers had significantly higher intakes of all other micronutrients, with a 23% higher intake of calcium, 32% higher iron, 24% higher thiamin, 30% higher riboflavin, 11% higher folate, 11% higher magnesium, and 9% higher intake of potassium than non-cereal breakfast consumers. Breakfast cereal consumers had a 9% lower daily intake of sodium compared with non-cereal breakfast consumers.

Comparing MPS and PS cereal consumers, there were no significant differences in total daily energy and nutrient intakes, including total, added, and free sugars, apart from folate, for which PS cereal consumers had a 14% higher intake than MPS cereal consumers. There were no significant differences in total daily intakes of added and free sugars between MPS, PS15, and PS30 cereal consumers.

In the models that included the breakfast category (breakfast skippers vs. non-cereal breakfast consumers vs. breakfast cereal consumers), out of the variables related to sex, age group, energy intake, BMI *z-*score category, and the interaction of sex and age group, energy intake was the only significant variable that influenced added and free sugars intake (*p* < 0.001). The R-squared value for the models was 37.7% for added sugars, and 39.7% for free sugars. In the models that included breakfast cereal type (MPS cereal vs. PS cereal consumers), out of the variables related to sex, age group, energy intake, BMI *z-*score category, and the interaction of sex and age group, energy intake was the only significant variable that influenced added and free sugars intake (*p* < 0.001). The R-squared value for the models was 41.8% for added sugars, 43.8% for free sugars.

### 3.3. Nutrient Contributions from Breakfast Cereal

Breakfast cereal contributed 10% of the total daily energy among breakfast cereal consumers, as well as 8% of protein, 4% of total fat, 14% of carbohydrates, and 7% of total sugars (Table 4). For micronutrients, breakfast cereal contributed 8% of total daily sodium; between 7% and 10% of the minerals calcium, magnesium, and potassium; 35% of iron; and between 17% and 37% of the B Vitamins thiamin, riboflavin, niacin, and folate. Among PS cereal consumers, breakfast cereals contributed significantly more to folate and calcium intakes compared with MPS cereal consumers. Breakfast cereal contributed significantly more among MPS cereal consumers to daily intakes of protein, total and saturated fat, dietary fibre, niacin, magnesium, and potassium compared with PS cereal consumers. There was no significant difference in the contribution from breakfast cereal to carbohydrate, iron, thiamin, riboflavin, or sodium between MPS and PS cereal consumers.

The contribution of breakfast cereal to the total daily intake of added, free, and total sugars was significantly higher among PS cereal consumers than MPS cereal consumers (Table 4). Among MPS cereal consumers, breakfast cereal contributed significantly less to total daily added and free sugars than among PS15 and PS30 consumers (4.5 ± 0.6% of total daily added sugars and 3.9 ± 0.5% of total daily free sugars among MPS cereal consumers, compared with 22.1 ± 0.9% of added sugars and 18.5 ± 0.7% of free sugars among PS15 consumers, and 22.5 ± 1.8% of added sugars and 21.5 ± 1.4% of free sugars among PS30 consumers).

### 3.4. Sugars Contributions from the Breakfast Cereal Bowl

The total added and free sugars content of the cereal and the cereal bowl differed significantly by type of pre-sweetened cereal consumer. Among MPS consumers, the cereal contributed 1.0 ± 0.2 g of added sugars and 1.2 ± 0.2 g of free sugars, which was significantly less than PS15 consumers (7.7 ± 0.3 g of added sugars, 8.4 ± 0.3 g of free sugars) and PS30 consumers (10.5 ± 0.5 g of added sugars, 12.3 ± 0.5 g of free sugars). When food and beverage additions (i.e., milk, honey, fruit) to the bowl are included, the gap between added sugars in the bowl for MPS consumers and PS consumers becomes smaller, though still significantly lower for MPS consumers. The breakfast cereal bowl as a whole contributed, for MPS consumers, 4.3 ± 0.3 g of added sugars and 6.2 ± 0.3 g of free sugars; for PS15 cereal consumers, 7.8 ± 0.4 g and 8.6 ± 0.5 g; and for PS30 cereal consumers, 10.3 ± 0.7 g and 12.0 ± 0.9 g (*p* < 0.001). 

### 3.5. BMI z-Score and Waist:Height Ratio 

There was no significant difference in prevalence of overweight and obesity, BMI *z-*score, and waist-to-height ratio between breakfast cereal consumers, non-cereal consumers, and breakfast skippers (Table 5). When comparing MPS and PS cereal consumers, there was also no significant difference in prevalence of overweight and obesity, BMI *z-*score, or waist-to-height ratio among children and adolescents.

## 4. Discussion

A total of 91% of Australian children and adolescents aged 2–18 years consumed breakfast. Among these children, just under half had a cereal breakfast, which was predominantly a ready-to-eat and a minimally pre-sweetened cereal, compared to a non-cereal breakfast, which was bread and spread-based. Breakfast cereal consumers had greater total daily fibre and micronutrient intakes and lower sodium intakes than non-cereal consumers, and no difference was found in nutrient intakes by the total sugars content of the breakfast cereal consumed, except for daily folate, which was higher among pre-sweetened cereal consumers. Minimally pre-sweetened cereal consumers were more likely to add sugar to their cereal bowl than pre-sweetened consumers, but despite this, they had significantly less added and free sugars in their cereal bowl. Minimally pre-sweetened cereal consumers also had a lower contribution of added and free sugars from their breakfast cereal, but similar total daily added and free sugars intakes, and a higher contribution to daily dietary fibre and magnesium, but lower contribution to daily folate. The study found no significant differences in anthropometric measures across breakfast and cereal categories.

Compared with results from the last nationally representative nutrition survey among children and adolescents in Australia, the prevalence of breakfast skipping was higher in this 2011–2012 survey at 9% compared with 4% in the previous 2007 survey [5]. In both surveys, the prevalence of breakfast skipping increased with age, and was high among adolescents, a finding consistent across studies in Canada [7], the USA [19], New Zealand [20], and Europe [21,22]. In Australia, the prevalence of breakfast cereal consumption was lower in 2011–2012 at 45% compared with 66% in 2007; breakfast cereal consumption also decreased with age. In 2011–2012, almost twice as many 2–3-year old consumed breakfast cereal compared with 14–18-year old. The decrease in prevalence of breakfast and cereal consumption across surveys may be partially accounted for by the inclusion of 17- and 18-year old in this 2011–2012 analysis, whereas the 2007 survey only had participants up to 16 years of age. 

Breakfast consumption and the type of breakfast consumed were associated with daily nutrient intakes in this study population. Breakfast cereal consumers had the lowest total fat and sodium intakes, and the highest intakes of dietary fibre and key micronutrients, including the shortfall nutrients calcium and iron. This finding is consistent with two systematic reviews on the benefits of breakfast cereal consumption [2,23] and more recent cross-sectional studies [5,24,25,26]. This is particularly relevant for Australian adolescents, who have increased nutritional requirements, yet are the most likely age group to skip breakfast and the least likely to have breakfast cereal for breakfast. A large percentage of Australian adolescents also are not meeting their targets for calcium and magnesium, and among girls, for iron [27]. Research shows that compared with non-cereal consumers, breakfast cereal consumers tend to have better diets throughout the day, as measured by the Healthy Eating Index score [28].

The types of foods and beverages consumed at breakfast differed between breakfast cereal and non-cereal breakfast consumers, and may explain the healthier daily nutrient intakes of breakfast cereal consumers. A recent study among a nationally representative sample of Mexican children aged 4–13 years reported that children in the cereal and milk breakfast dietary pattern had the highest intakes of B vitamins (except folate), vitamin D, zinc, and iron at both breakfast and across the day [25]. In a recent study among European adolescents, a RTE cereal breakfast was associated with higher intakes of dairy milk or yoghurt, and greater intakes of dietary fibre, B vitamins, calcium, magnesium and phosphorus, compared with a bread-based breakfast or other breakfast [24]. Greater milk intake among breakfast cereal consumers may partially explain the better nutrient profile of breakfast cereal consumers compared with non-cereal consumers, especially for key nutrients that dairy milk provides, such as calcium, magnesium, potassium, and riboflavin. 

In this study, dairy milk was consumed by 87% of cereal consumers compared with just 24% of non-cereal consumers. Cereal consumption has been shown to drive milk intake [2], and dairy milk is the top source of calcium in the diet of Australian children and adolescents, providing 27% of total daily calcium per capita [8]. A secondary analysis of the 2007 Australian National Children’s Nutrition and Physical Activity Survey also found dairy food intake at the first eating occasion of the day to be important for total dairy food intake, with a 129% greater total daily intake of dairy foods for children who consumed dairy foods at the first eating occasion of the day [29]. Since more than 95% of 12- to 18-year-old fell short of national recommendations for milk, yoghurt, cheese and alternatives [30], breakfast cereal may be an important driver of consumption of these foods.

Breakfast cereals and breads are important sources of grain foods; however, only 56% of non-cereal breakfast consumers consumed bread at breakfast, which would impact on daily intake of both whole grains and dietary fibre. In Australia, the 2007 Australian National Children’s Nutrition and Physical Activity Survey showed that RTE cereals were a significant predictor of total daily fibre intake [31]. Evidence from national surveys of Australian adolescents [32] and U.S. children [33] also show that daily fibre intake is higher when breakfast is consumed, particularly breakfast containing RTE cereal, than when it is skipped, suggesting that low dietary fibre intakes at breakfast are not made up over the remainder of the day.

Among Australian children, the majority (87%) of breakfast cereal consumers were exclusive consumers of ready-to-eat (RTE) breakfast cereal. Australian RTE cereals can be fortified with micronutrients such as B vitamins, iron, and calcium [34], which can add to the contribution that breakfast cereal makes to intakes of key nutrients and to the daily intake differences observed between cereal and non-cereal breakfast consumers. Breakfast cereals contributed over a third of daily iron intakes, over 20% of daily folate intakes, and between 7–14% of daily calcium. Few differences in the contribution from the cereal were observed based on the total sugars content of the breakfast cereal. Among PS cereal (>15% total sugars) consumers, the cereal contributed more to folate and calcium than among MPS cereal consumers, and resulted in greater intake of dietary folate. This may be due to the fortification of these cereals with folic acid. However, no other differences in daily nutrients were found. Similarly, American children and adolescents who had a PS cereal (>17% total sugars) had higher daily intakes of folate, but also higher calcium intakes [11]. A recent randomized, double-blind, placebo controlled interventional trial among adolescent girls aged 16–19 years reported that the consumption of 50 g of fortified cereal and low-fat milk for 12 weeks resulted in significant increases to daily intakes of B vitamins, including folate, iron and vitamin D, and that it also translated to significant increases in the nutritional status of riboflavin, vitamin B_12_, folate, and iron, compared with those who received an unfortified RTE cereal [35]. Among children and adolescents in the USA, RTE cereals contributed to a higher folate and vitamin B_12_ status, but a very small percentage of children and adolescents were deficient in these vitamins, primarily due to mandatory folate fortification [36]. A systematic review on the health benefits of RTE cereals reported that among children, RTE cereal consumption decreased the prevalence of nutrient inadequacies for vitamin A, calcium, folate, magnesium and zinc [23]. Thus, the fortification of breakfast cereal seems to be an effective method of delivering essential micronutrients.

The finding that the sugars content of the cereal did not negatively affect total daily nutrient intakes is consistent with an Australian modelling study of carbohydrate-rich foods that found the sugars content of a breakfast cereal to be a poor predictor of its nutrient density score [9].

There has been much focus on the sugar content of breakfast cereals [37]. This study showed that minimally pre-sweetened cereal was the most popular amongst Australian children. The majority (62%) of breakfast cereal consumers had a minimally pre-sweetened cereal, and only 8% had cereal with greater than 30% total sugars. This is in contrast to a study among 6–18-year old in the USA, where 64% of children who were breakfast cereal consumers consumed breakfast cereals with greater than 33% total sugars [11]. This highlights the importance of putting data into context when reporting on dietary habits and the health of populations. 

Compared to non-cereal breakfast consumers, breakfast cereal consumers had a higher total daily sugars intake, a finding supported by two systematic reviews [2,23]. This observation is likely influenced by the contribution to total sugars intakes from fruit and milk consumed with the cereal. In this study, daily added and free sugars intakes were not significantly different across breakfast categories or across breakfast cereals with differing total sugars content. Whilst there were differences between the contribution to total added/free sugars between MPS, PS15, and PS30 cereals, there were no significant differences in total daily added/free sugars intakes, which demonstrates that the rest of the day also needs to be taken into account when recommending measures to limit added/free sugars intakes. 

MPS cereal consumers had the least added and free sugars in their cereal bowl despite adding more sugar to the bowl, and the cereal contributed four times less to daily added and free sugars intakes, and more to daily dietary fibre than among PS consumers. Therefore, the recommendation to choose a MPS cereal and to limit discretionary cereals, or those that have >30 g/100 g of total sugars, is supported by this research, and in line with the Australian Dietary Guidelines [38], which recommend limiting the intake of discretionary foods, and choosing grain foods that are higher in dietary fibre, whole grains, and lower in added sugars. In addition, experimental research from the U.S. has shown that when presented with a lower sugars cereal, children still reported high acceptability ratings for that cereal [39].

The contribution of the breakfast occasion to total daily added sugars based on the 2011–2012 National Nutrition and Physical Activity Survey in Australia ranges between 15% among 14–18-year old and 19% among 2–3-year old, compared to 52% from non-main meal-eating occasions [40]. The authors of that study suggest that while the re-formulation of breakfast foods that are high in sugars (such as high-sugar breakfast cereals) is a possible strategy to lower the population’s intake of added sugars, given the high contribution of non-main meals to added sugars intake, it would be less effective than targeting a reduction in the intake of low-nutrient dense, high-sugar discretionary foods and beverages, which unlike breakfast cereal consumption, provide little to no nutritional value. 

Lastly, the study found no associations between breakfast nor type of breakfast consumed and BMI *z-*score or waist to height ratio among Australian children and adolescents. In Australia, a 20-year study on a national sample of children and adolescents found that eating breakfast in both childhood and adulthood was associated with a smaller waist circumference and improved cardiometabolic health markers when compared with those who skipped breakfast at both time points [41]. In addition, greater breakfast frequency has been shown to predict less weight gain over five years in a cohort of adolescents from the USA [42]. For breakfast cereal in particular, a systematic review and meta-analysis of 14 studies determined that breakfast cereal consumption was associated with a lower BMI and reduced likelihood of being overweight in children and adolescents, when compared with the consumption of other breakfasts, or breakfast skipping [43]. Thus, there is no evidence that any recommendations to consume breakfast or breakfast cereal would be associated with negative adiposity outcomes.

This study used usual intake data based on two days of recall to determine the prevalence of breakfast cereal consumption. One 24-h recall was used to calculate mean intakes, which does not reflect usual intake; however, mean intakes do not substantially differ from usual intakes. As with any study of a cross-sectional nature, it is not possible to determine causal relationships between breakfast choice or cereal type and nutrient intakes. However, by examining foods consumed by breakfast type and the contribution of different breakfast cereals to daily nutrient intakes, including added sugars, the study was able to characterise the relationship between breakfast choice and nutrient intakes in more detail, in the largest available, nationally representative survey sample.

## 5. Conclusions

Breakfast cereal consumers had a superior micronutrient profile and dietary fibre intake compared with skippers and non-cereal breakfast consumers. Australian adolescents, who are at the greatest risk of not meeting nutrient targets for key nutrients, including calcium and magnesium, had the highest prevalence of breakfast skipping, and the lowest prevalence of breakfast cereal consumption. Breakfast cereal consumers had a higher total daily sugars intake than non-cereal consumers, but there was no difference in daily added and free sugars intakes, indicating that other foods may play a larger role in added and free sugars intakes than breakfast cereal. The nutrient profile of breakfast cereal consumers compared with non-cereal breakfast consumers and breakfast skippers, combined with the lack of association with anthropometric measures, provides strong evidence for recommending breakfast cereal at the breakfast meal.

## Figures and Tables

**Table 1:** The weighted percentage of children and adolescents, 2–18 years by breakfast * consumption and breakfast cereal type based on the 2011–2012 National Nutrition and Physical Activity Survey (*n* = 2812).

	Total	Breakfast Category						Breakfast Cereal Consumers					
		Skippers		Non-Cereal Consumers		Breakfast Cereal Consumers		MPS		PS15		PS30	
	N	N	% (95% CI)	N	% (95% CI)	N	% (95% CI)	N	% (95% CI)	N	% (95% CI)	N	% (95% CI)
**All children**	2812	241	8.6 (7.5, 9.6)	1302	46.3 (44.5, 48.1)	1269	45.1 (43.3, 47.0)	784	61.8 (59.1, 64.5)	385	30.3 (27.8, 32.8)	100	7.9 (6.4, 9.4)
Boys	1435	127	8.9 (7.4, 10.3)	622	43.3 (40.8, 45.9)	686	47.8 (45.2, 50.4)	424	61.9 (58.3, 65.5)	212	30.9 (27.4, 34.3)	50	7.2 (5.3, 9.2)
Girls	1377	114	8.3 (6.8, 9.7)	680	49.4 (46.7, 52.0)	584	42.4 (39.8, 45.0)	360	61.7 (57.7, 65.6)	173	29.7 (26.0, 33.4)	50	8.6 (6.4, 10.9)
**Age (years)**													
2–3	331	4	1.2 (0.0, 2.3)	134	40.5 (35.2, 45.8)	193	58.3 (53.0, 63.6)	136	70.5 (64.0, 76.9)		25.9 (19.7, 32.1)	7	3.7 (1.0, 6.3)
4–8	809	15	1.9 (1.0, 2.8)	333	41.2 (37.8, 44.6)	460	56.9 (53.5, 60.3)	297	64.4 (60.0, 68.8)	126	27.4 (23.3, 31.5)	38	8.2 (5.7, 10.7)
9–13	900	76	8.4 (6.6, 10.3)	456	50.7 (47.5, 54.0)	367	40.8 (37.6, 44.0)	214	58.3 (53.2, 63.3)	126	34.4 (29.5, 39.2)	27	7.3 (4.7, 10.0)
14–18	773	146	18.9 (16.1, 21.6)	378	49.0 (45.4, 52.5)	249	32.2 (28.9, 35.5)	138	55.4 (49.2, 61.6)	82	33.1 (27.3, 39.0)	28	11.4 (7.5,15.4)

Abbreviations: CI—confidence interval; MPS—minimally pre-sweetened with <15% total sugars; PS15—presweetened with ≥15% <30% total sugars; PS30—pre-sweetened with ≥30% total sugars. * Skippers did not report a “breakfast” eating occasion. Breakfast cereal consumers had any food from the sub-major food groups “breakfast cereals, ready to eat” or “breakfast cereals, hot porridge style”, either at any time of day with a “breakfast” eating occasion, or between 5.30 a.m. and 9.30 a.m. with an “extended consumption” eating occasion. Non-cereal consumers reported “breakfast” eating occasions, but were not breakfast cereal consumers. MPS cereal consumers were exclusive consumers of a cereal with <15% total sugars. Pre-sweetened cereal consumers had a PS15 cereal or a PS30.

**Table 2:** Top 10 sub-major food groups consumed at breakfast ranked by prevalence of consumption amongst cereal and non-cereal consumers aged 2–18 years based on the 2011–2012 National Nutrition and Physical Activity Survey (*n* = 2812).

	Breakfast Consumers *					
Rank	Breakfast Cereal (*n* = 1269)			Non-Cereal (*n* = 1302)		
	Sub-Major Food Group	% Consumers	Median Grams Per Consumer	Sub-Major Food Group	% Consumers	Median Grams Per Consumer
1	Breakfast cereals, ready to eat	89.2%	34	Regular breads, and bread rolls (plain/unfilled/untopped varieties)	56.3%	54
2	Dairy milk (cow, sheep, and goat)	86.5%	162	Dairy milk (cow, sheep and goat)	24.5%	206
3	Sugar, honey, and syrups	31.9%	7	Margarine and table spreads	22.7%	5
4	Fruit and vegetable juices, and drinks	13.3%	235	Fruit and vegetable juices, and drinks	19.6%	252
5	Breakfast cereals, hot porridge style	11.2%	202	Yeast, and yeast vegetable or meat extracts	17.9%	6
6	Waters, municipal and bottled, unflavoured	10.8%	250	Butters	14.1%	5
7	Regular breads, and bread rolls (plain/unfilled/untopped varieties)	10.0%	35	Other beverage flavourings and prepared beverages	12.2%	15
8	Other beverage flavourings and prepared beverages	5.2%	8	Sugar, honey, and syrups	11.0%	8
9	Margarine and table spreads	4.5%	5	Waters, municipal and bottled, unflavoured	10.8%	250
10	Tropical and subtropical fruit	2.8%	98	Jam and lemon spreads, chocolate spreads, sauces	10.1%	10

* Breakfast cereal consumers had any food from the sub-major food groups “breakfast cereals, ready to eat” or “breakfast cereals, hot porridge style”, either at any time of day with a “breakfast” eating occasion, or between 5.30 a.m. and 9.30 a.m. with an “extended consumption” eating occasion. Non-cereal consumers reported “breakfast” eating occasions, but were not cereal consumers.

**Table 3:** Total daily energy and nutrient intakes by categories of breakfast consumers * and breakfast cereal type among 2–18-year-old based on the 2011–2012 National Nutrition and Physical Activity Survey (*n* = 2812).

Nutrient ^†^	Breakfast Category			Breakfast Cereal Consumers	
	Breakfast Skippers (*n* = 241)	Non-Cereal Breakfast Consumers (*n* = 1302)	Breakfast Cereal Consumers (*n* = 1269)	MPS (*n* = 784)	PS (*n* = 485)
	Daily Nutrient Intake (mean ± SE)				
Energy (kJ)	6832 ± 202 ^a^	7632 ± 86 ^a,b^	7798 ± 86 ^b^	7847 ± 108	7716 ± 139
Protein (g)	74.0 ± 1.7	76.4 ± 0.8	76.7 ± 0.8	75.6 ± 1.0	78.6 ± 1.2
Total fat (g)	78.3 ± 1.3 ^a^	71.7 ± 0.6 ^b^	67.1 ± 0.6 ^c^	67.1 ± 0.7	66.9 ± 0.9
*Saturated fat* (g)	32.1 ± 0.7 ^a^	29.7 ± 0.3 ^a,b^	29.0 ± 0.3 ^b^	29.4 ± 0.4	28.3 ± 0.5
Carbohydrate (g)	225 ± 3.3 ^a^	237 ± 1.5 ^a^	246 ± 1.6 ^b^	247 ± 1.9	244 ± 2.3
Total Sugars (g)	111 ± 3.3 ^a,b^	111 ± 1.5 ^a^	119.4 ± 1.6 ^b^	119 ± 1.9	120 ± 2.4
Added sugars ** (g)	63.0 ± 2.7	58.4 ± 1.1	53.5 ± 1.1	52.8 ± 1.4	54.7 ± 1.8
Free sugars ** (g)	72.7 ± 2.8	66.2 ± 1.2	62.7 ± 1.2	62.0 ± 1.5	64.0 ± 1.9
Dietary fibre (g)	16.8 ± 0.6 ^a^	18.6 ± 0.3 ^a^	21.7 ± 0.3 ^b^	21.7 ± 0.3	21.7 ± 0.4
Niacin (equivalents) (mg)	31.7 ± 0.9	33.4 ± 0.4	34.6 ± 0.4	34.6 ± 0.5	34.5 ± 0.7
Iron (mg)	7.8 ± 0.3 ^a^	8.4 ± 0.1 ^a^	11.7 ± 0.1 ^b^	11.4 ± 0.2	12.3 ± 0.2
Thiamin (mg)	1.2 ± 0.1 ^a^	1.5 ± 0.0 ^b^	1.9 ± 0.0 ^c^	1.9 ± 0.0	1.9 ± 0.1
Riboflavin (equivalents) (mg)	1.4 ± 0.1 ^a^	1.7 ± 0.0 ^a^	2.3 ± 0.0 ^b^	2.3 ± 0.0	2.2 ± 0.1
Folate (µg)	433 ± 25 ^a^	610 ± 11 ^b^	683 ± 12 ^c^	648 ± 14 ^a^	744 ± 18 ^b^
Calcium (mg)	721 ± 29 ^a^	740 ± 13 ^a^	930 ± 14 ^b^	908 ± 16	967 ± 20
Sodium (mg)	2430 ± 68 ^a,b^	2405 ± 32 ^a^	2190 ± 32 ^b^	2139 ± 39	2278 ± 49
Magnesium (mg)	226 ± 4.9 ^a^	248 ± 2.3 ^b^	278 ± 2.3 ^c^	281 ± 2.8	273 ± 3.5
Potassium (mg)	2397 ± 56 ^a,b^	2348 ± 26 ^a^	2557 ± 26 ^b^	2536 ± 32	2592 ± 40

Abbreviations: SE—standard error; MPS—minimally pre-sweetened with <15% total sugars; PS—presweetened with ≥15% total sugars. ^†^ Energy means were adjusted for sex, age group, the interaction of sex and age group using general linear models. Nutrient means were adjusted for sex, age group, the interaction of sex and age group, energy intake, and BMI *z-*score category using general linear models. * Breakfast skippers did not report a “breakfast” eating occasion. Breakfast cereal consumers had any food from the sub-major food groups “breakfast cereals, ready to eat” or “breakfast cereals, hot porridge style”, either at any time of day with a “breakfast” eating occasion, or between 5.30 a.m. and 9.30 a.m. with an “extended consumption” eating occasion. Non-cereal consumers reported “breakfast” eating occasions but were not breakfast cereal consumers. MPS cereal consumers were exclusive consumers of a cereal with <15% total sugars. PS cereal consumers were consumers of a cereal with ≥15% total sugars. ** Added and free sugars intake were further calculated for PS consumers by PS15 and PS30 consumers. Added sugars for PS15 consumers = 52.0 ± 2.0 g, PS30 = 65.4 ± 4.0 g, no significant difference between MPS consumers for either group. Free sugars for PS15 consumers = 60.4 ± 2.1 g, PS30 = 77.8 ± 4.1 g, no significant difference between MPS consumers for either group. Different superscripts a, b, c denotes significant difference between groups (*p* < 0.001) by post hoc, Bonferroni.

**Table 4:** The mean contribution of breakfast cereal to total daily energy nutrient intakes by pre-sweetening * of the breakfast cereal in breakfast cereal consumers aged 2–18 years based on the 2011–2012 National Nutrition and Physical Activity Survey (*n* = 2812).

Nutrient ^†^	Breakfast Cereal Consumers (*n* = 1269)	Breakfast Cereal Consumers	
		MPS (*n* = 784)	PS (*n* = 485)
	Contribution to Total Daily Intake (% ± SE)		
Energy	9.9 ± 0.2	10.3 ± 0.3	9.6 ± 0.4
Protein	8.4 ± 0.3	9.3 ± 0.3 ^a^	7.0 ± 0.4 ^b^
Total fat	3.6 ± 0.2	4.1 ± 0.3 ^a^	2.9 ± 0.4 ^b^
*Saturated fat*	2.6 ± 0.2	3.3 ± 0.3 ^a^	1.4 ± 0.4 ^b^
Total sugars	6.6 ± 0.3	4.3 ± 0.4 ^a^	11.3 ± 0.5 ^b^
*Added sugars*	11.6 ± 0.7	4.5 ± 0.6 ^a^	22.2 ± 0.8 ^b^
*Free sugars*	10.0 ± 0.6	3.9 ± 0.5 ^a^	19.1 ± 0.7 ^b^
Carbohydrate	13.8 ± 0.3	13.8 ± 0.4	13.9 ± 0.5
Dietary fibre	18.5 ± 0.5	21.2 ± 0.6 ^a^	14.0 ± 0.8 ^b^
Niacin	17.1 ± 0.4	18.7 ± 0.5 ^a^	14.0 ± 0.7 ^b^
Iron	35.1 ± 0.6	35.3 ± 0.8	34.9 ± 1.0
Thiamin	37.1 ± 0.8	35.6 ± 1.0	38.8 ± 1.3
Riboflavin	25.6 ± 0.6	24.7 ± 0.7	25.9 ± 0.9
Folate	24.7 ± 0.7	21.8 ± 0.9 ^a^	30.7 ± 1.1 ^b^
Calcium	9.9 ± 0.5	7.3 ± 0.7 ^a^	14.2 ± 0.8 ^b^
Sodium	7.5 ± 0.2	7.5 ± 0.3	7.9 ± 0.4
Magnesium	14.5 ± 0.4	16.7 ± 0.5 ^a^	10.9 ± 0.6 ^b^
Potassium	6.9 ± 0.3	8.0 ± 0.4 ^a^	5.2 ± 0.4 ^b^

Abbreviations: SE—standard error; MPS—minimally pre-sweetened, PS—pre-sweetened with ≥15% total sugars. ^†^ Mean energy contribution adjusted for sex, age group, the interaction of sex and age group, and BMI *z-*score category. Mean nutrient contribution was adjusted for energy intake, sex, age group, the interaction of sex and age group, and BMI *z-*score category. * MPS cereal consumers were exclusive consumers of a cereal with <15% total sugars. PS cereal consumers were consumers of a cereal with ≥15% total sugars. Different superscripts a, b, denotes significant difference between groups (*p* < 0.001) by post hoc, Bonferroni.

**Table 5:** The weighted percentage of children and adolescents, 2–18 years in each zBMI category by breakfast. Consumption and breakfast cereal type based on the 2011–2012 National Nutrition and Physical Activity Survey (*n* = 2812).

zBMI Category	Total		Breakfast Category						Breakfast Cereal Consumers			
			Skippers *		Non-Cereal Consumers		Breakfast Cereal Consumers		MPS		PS	
	*n* ^+^	%	*n*	%	*n*	%	*n*	%	*n*	%	*n*	%
Normal weight	1524	68.5	134	69.8	707	67.8	683	68.9	411	67.3	272	71.4
At risk for overweight	285	12.8	22	11.5	137	13.1	126	12.7	88	14.4	39	10.2
Overweight	417	18.7	36	18.8	199	19.1	182	18.4	112	18.3	70	18.4

Abbreviations: MPS—minimally pre-sweetened with <15% total sugars; PS—pre-sweetened with ≥15% total sugars. * Skippers did not report a “breakfast” eating occasion. Breakfast cereal consumers had any food from the sub-major food groups “breakfast cereals, ready to eat” or “breakfast cereals, hot porridge style”, either at any time of day with a “breakfast” eating occasion, or between 5.30 a.m. and 9.30 a.m. with an “extended consumption” eating occasion. Non-cereal consumers reported a “breakfast” eating occasion, but were not breakfast cereal consumers. MPS cereal consumers were exclusive consumers of a cereal with <15% total sugars. PS cereal consumers were consumers of a cereal with ≥15% total sugars. ^+^ Does not include children who did not have height and/or weight measurements taken.

## Supplementary Materials

The following are available online at http://www.mdpi.com/2072-6643/9/10/1045/s1, Table S1: The weighted percentage of breakfast cereal consumers among children and adolescents, 2–18 years, by type of breakfast cereal * consumed, Table S2: The contribution of the breakfast cereal to total daily nutrient intakes by type of breakfast cereal in breakfast cereal consumers *, Table S3: Total daily energy and nutrient intake by cereal type in breakfast cereal consumers *.
