# Supplementary material for: Breakfast and Breakfast Cereal Choice and Its Impact on Nutrient and Sugar Intakes and Anthropometric Measures among a Nationally Representative Sample of Australian Children and Adolescents
Source: Nutrients. 2017 Sep 21;9(10):1045. doi: 10.3390/nu9101045 (PMC5691662; doi:10.3390/nu9101045)
Supplement: Supplementary file 1 [file nutrients-09-01045-s001.zip › nutrients-220668-supplementary.pdf]

Table S1. The weighted percentage of breakfast cereal consumers among children and adolescents, 2-18years, by type of breakfast cereal * consumed.

	Breakfast Cereal Consumers							
	RTE		Muesli		Hot-Porridge Style		Mixed	
	n	% (95% CI)	n	% (95% CI)	n	% (95% CI)	n	% (95% CI)
All children	1103	86.9 (85.1, 88.8)	18	1.5 (0.8, 2.1)	137	10.8 (9.1, 12.5)	10	0.8 (0.3, 1.3)
Sex								
Boys	613	89.5 (87.2, 91.8)	7	1.0 (0.2, 1.7)	58	8.5 (6.4, 10.6)	7	1.1 (0.3, 1.8)
Girls	490	83.9 (80.9, 86.9)	12	2.0 (0.9, 3.2)	79	13.5 (10.8, 16.3)	3	0.5 (-0.1, 1.1)
Age group								
2-3 years	166	86.0 (81.1, 90.9)	0	0.2 (-0.4, 0.8)	21	11.0 (6.6, 15.4)	5	2.8 (0.5, 5.2)
4-8 years	400	86.9 (83.9, 90.0)	5	1.1 (0.1, 2.1)	54	11.7 (8.7, 14.6)	1	0.3 (-0.2, 0.8)
9-13 years	323	87.9 (84.6, 91.3)	2	0.5 (-0.2, 1.3)	39	10.6 (7.4, 13.7)	3	1.0 (0.0, 1.9)
14-18 years	214	86.1 (81.8, 90.4)	11	4.4 (1.9, 7.0)	24	9.5 (5.8, 13.1)	0	0.0 (0.0, 0.0)

Abbreviations: CI—confidence interval; RTE—ready-to-eat. * RTE cereal consumers were exclusive consumers at breakfast of sub-major food groups 'breakfast cereals, ready to eat', apart from 12514007–12514024 and 12515027. Muesli consumers were exclusive consumers at breakfast of any of food groups 12514007–12515024 or 12515027. Hot-porridge style cereal consumers were exclusive consumers at breakfast of the food group 'breakfast cereals, hot porridge style'. Mixed cereal consumers had more than one type of cereal at breakfast.

Table S2. The contribution of the breakfast cereal to total daily nutrient intakes by type of breakfast cereal in breakfast cereal consumers *.

**Nutrient** †	**Breakfast Cereal Consumers**		
	**RTE (*n* = 1103)**	**Muesli (*n* = 18)**	**Hot-Porridge Style (*n* = 137)**
	**Contribution to Total Daily Intake (% ± SE)**		
Energy	9.1 ± 0.3 [a]	16.0 ± 1.6 [b]	17.1 ± 0.6 [b]
Protein	7.2 ± 0.3 [a]	10.1 ± 1.7 [a]	18.4 ± 0.6 [b]
Total fat	2.2 ± 0.2 [a]	12.9 ± 1.1 [b]	14.9 ± 0.4 [b]
Saturated fat	1.1 ± 0.2 [a]	7.5 ± 1.2 [b]	14.1 ± 0.4 [c]
Total sugars	5.8 ± 0.4 [a]	18.3 ± 2.2 [b]	13.7 ± 0.8 [b]
Added sugars	12.6 ± 0.8	14.3 ± 5.2	5.9 ± 1.9
Free sugars	10.6 ±0.7	14.4 ± 4.3	4.8 ± 1.6
Carbohydrate	13.1 ± 0.3 [a]	23.4 ± 2.1 [b]	18.3 ± 0.7 [b]

Dietary fibre	17.8 ± 0.6 [a]	29.0 ± 3.7 [a,b]	24.4 ± 1.3 [b]
Niacin	17.3 ± 0.5	12.4 ± 3.1	14.5 ± 1.1
Iron	36.9 ± 0.7 [a]	30.5 ± 4.4 [a,b]	21.9 ± 1.6 [b]
Thiamin	39.7 ± 0.9 [a]	22.4 ± 5.3 [a,b]	14.9 ± 1.9 [b]
Riboflavin	25.5 ± 0.7	11.8 ± 4.2	23.4 ± 1.5
Folate	27.3 ± 0.8 [a]	9.0 ± 4.8 [b]	8.2 ± 1.7 [b]
Calcium	7.7 ± 0.5 [a]	6.7 ± 3.4 [a]	27.0 ± 1.2 [b]
Sodium	8.0 ± 0.3	2.7 ± 1.9	5.5 ± 0.7
Magnesium	13.0 ± 0.4 [a]	28.4 ± 2.7 [b]	26.2 ± 1.0 [a,b]
Potassium	5.3 ± 0.3 [a]	12.9 ± 1.6 [b]	19.1 ± 0.6 [b]

Abbreviations: CI—confidence interval; RTE—ready-to-eat. * RTE cereal consumers were exclusive consumers at breakfast of sub-major food groups 'breakfast cereals, ready to eat', apart from 12514007–12514024 and 12515027. Muesli consumers were exclusive consumers at breakfast of any of food groups 12514007–12515024 or 12515027. Hot-porridge style cereal consumers were exclusive consumers at breakfast of the food group 'breakfast cereals, hot porridge style'. † Mean energy contribution was adjusted for sex, age group, the interaction of sex and age group, and BMI z-score category. Mean nutrient contribution was adjusted for sex, age group, the interaction of sex and age group, energy intake, and BMI z-score category. Different superscripts a, b, c denotes significant difference between groups ($p < 0.001$) by post hoc, Bonferroni.

Table S3. Total daily energy and nutrient intake by cereal type in breakfast cereal consumers *.

**Nutrient †**	**Breakfast Cereal Consumers**		
	**RTE (*n* = 1103)**	**Muesli (*n* = 18)**	**Hot-Porridge Style (*n* = 137)**
	**Daily Nutrient Intake (Mean ± SE)**		
Energy (kJ)	7746 ± 92	9604 ± 764	7942 ± 254
Protein (g)	76.2 ± 0.9	93.8 ± 6.2	78.0 ± 2.2
Total fat (g)	67.0 ± 0.6	78.5 ± 4.5	67.0 ± 1.6
Saturated fat (g)	29.3 ± 0.4	28.8 ± 2.6	27.5 ± 0.9
Total sugars (g)	120 ± 1.7	94.4 ± 12	118 ± 4.2
Added sugars (g)	55.6 ± 1.6	23.4 ± 9.8	49.5 ± 3.5
Free sugars (g)	64.3 ± 1.6	32.2 ± 10.3	57.7 ± 3.7
Carbohydrate (g)	247 ± 1.6	200 ± 12	244 ± 4.1
Dietary fibre (g)	21.4 ± 0.3	28.2 ± 2.1	22.4 ± 0.7
Niacin (mg)	34.9 ± 0.5	45.1 ± 3.3	30.5 ± 1.2
Iron (mg)	12.0 ± 0.1 [a]	12.2 ± 1.0 [a,b]	9.3 ± 0.4 [b]

Thiamin (mg)	2.0 ± 0.04 [a]	2.5 ± 0.3 [a,b]	1.3 ± 0.1 [b]
Riboflavin (mg)	2.4 ± 0.04 [a]	1.52 ± 0.3 [a,b]	1.9 ± 0.1 [b]
Folate (μg)	705 ± 12.3 [a]	583 ± 86 [a,b]	522 ± 31 [b]
Calcium (mg)	936 ± 14	645 ± 100	942 ± 36
Sodium (mg)	2221 ± 34 [a,b]	3001 ± 240 [a]	1857 ± 86 [b]
Magnesium (mg)	274 ± 2.4 [a]	357 ± 17 [b]	294 ± 6.1 [a,b]
Potassium (mg)	2534 ± 27.8	2919 ± 196	2577 ± 70

Abbreviations: CI— confidence interval; RTE—ready-to-eat. * RTE cereal consumers were exclusive consumers at breakfast of sub-major food groups 'breakfast cereals, ready to eat', apart from 12514007–12514024 and 12515027. Muesli consumers were exclusive consumers at breakfast of any of food groups 12514007–12515024 or 12515027. Hot-porridge style cereal consumers were exclusive consumers at breakfast of the food group 'breakfast cereals, hot porridge style'. Mixed cereal consumers had more than one type of cereal at breakfast. † Mean energy contribution was adjusted for sex, age group, the interaction of sex and age group, and BMI z-score category. Mean nutrient contribution was adjusted for energy intake, sex, age group, the interaction of sex and age group, and BMI z-score category. Different superscripts a, b, c denotes significant difference between groups ($p < 0.001$) by post hoc, Bonferroni.
